# The impact of scaling-up combination antiretroviral therapy on patterns of mortality among HIV-positive persons in British Columbia, Canada

**DOI:** 10.7448/IAS.18.1.20261

**Published:** 2015-10-07

**Authors:** Viviane Dias Lima, Oghenowede Eyawo, Huiting Ma, Lillian Lourenço, William Chau, Robert S Hogg, Julio SG Montaner

**Affiliations:** 1 British Columbia Centre for Excellence in HIV/AIDS, Vancouver, BC, Canada; 2 Division of AIDS, Department of Medicine, Faculty of Medicine, University of British Columbia, Vancouver, BC, Canada; 3 Faculty of Health Sciences, Simon Fraser University, Burnaby, BC, Canada

**Keywords:** PLHIV, mortality rate, life expectancy, standardized mortality ratios, clinical outcomes, treatment as prevention, British Columbia, Canada

## Abstract

**Introduction:**

Despite the tremendous improvements in survival, some groups of people living with HIV (PLHIV) continue to have lower survival rates than the overall HIV-positive population. Here, we characterize the evolving pattern of mortality among PLHIV in British Columbia since the beginning of the expansion of antiretroviral treatment in 2003.

**Methods:**

This retrospective cohort study included 3653 individuals ≥20 years old, who enrolled on treatment between January 1, 2003, and December 31, 2012, and were followed until December 31, 2013. All-cause mortality rates and standardized mortality ratios (SMRs) were calculated to compare mortality outcomes of PLHIV to the general population. Abridged life tables were constructed to estimate the life expectancy at age 20 years for PLHIV.

**Results:**

The overall crude mortality rate was 28.57 per 1000 person-years, the SMR was 3.22 and the life expectancy was 34.53 years. Interestingly, if we considered only individuals alive after the first year, the life expectancy increased to 48.70 years (41% increase). The SMRs for males and females decreased over time. Although females had higher SMRs in 2003 to 2008, this difference no longer existed in 2009 to 2011. There were also important differences in mortality outcomes for different clinical and demographical characteristics.

**Conclusions:**

Mortality outcomes of PLHIV who initiated antiretroviral treatment have dramatically improved over the last decade. However, there is still room for improvement and multilateral efforts should continue to promote early, sustained engagement of PLHIV on treatment so that the impact of treatment can be fully realized.

## Introduction

People living with HIV (PLHIV) across the globe have seen tremendous improvements in their overall survival, due in part to the introduction and increasingly widespread use of combination antiretroviral therapy (cART) [1,2]. In high-income countries like the United States, Canada and those in the European Union, HIV is increasingly being managed like a chronic infection. People living in such settings now have similar life expectancies as those observed in the general population [3–5]. However, some groups of PLHIV in these countries continue to have lower survival rates than the overall HIV-positive population [6], as a result of socio-economic constraints [7], poor engagement in HIV care and in treatment [8] and a lack of access to the healthcare system [9]. Further characterizing and identifying these subgroups should be a priority, so that the impact of cART in terms of decreasing morbidity, mortality and transmission can be fully optimized.

In the province of British Columbia (BC), Canada, there have been different cART expansion periods since 2003 [10]. However, only in 2010 was treatment as prevention (TasP) implemented in BC to maximize engagement of PLHIV along the HIV continuum of care to increase the likelihood of viral suppression and, therefore, decrease HIV-related morbidity and mortality as well as new HIV infections [11–14]. TasP in BC also aimed to eliminate significant health and social disparities among men and women living with the virus that have contributed to the high burden of HIV disease mortality. In BC, in 2011, the Public Health Agency of Canada estimated that 11,700 individuals were living with HIV [15]. They noted that the population subgroups more affected by HIV were men who have sex with men (42%), people who inject drugs (31%) and heterosexual individuals from countries where HIV is not endemic (19%).

The objective of this study was to characterize the evolving pattern of mortality among PLHIV in BC since the beginning of the expansion of antiretroviral treatment in 2003. In this population, we examined the heterogeneity of several mortality measures across gender, age and different clinical characteristics.

## Methods

### Data

PLHIV were eligible for this study if they were registered to receive cART from the BC Centre for Excellence in HIV/AIDS (BC-CfE) Drug Treatment Program. Since October 1992, the distribution of antiretrovirals in BC has been the responsibility of BC-CfE. Antiretroviral drugs are distributed to all PLHIV according to specific guidelines generated by the BC-CfE's Therapeutic Guidelines Committee [16]. These have remained consistent with those put forward by the International AIDS Society-USA [17–22].

PLHIV included in this analysis were cART naive, ≥20 years old, enrolled between January 1, 2003, and December 31, 2012, and followed until December 31, 2013. Treatment eligibility, options for initial cART regimen for treatment-naive adults and treatment monitoring were based on the HIV treatment guidelines between 2003 and 2012 [17–22]. In this study, individuals started cART typically consisting of two nucleoside reverse-transcriptase inhibitors as backbone, plus either a non-nucleoside reverse-transcriptase inhibitor (efavirenz or nevirapine) or a ritonavir-boosted protease inhibitor (lopinavir or atazanavir). They must also have had a CD4 count and plasma viral load measurement within six months of the initial cART date. All plasma viral load measurements in BC are centrally done at the St Paul's Hospital virology laboratory. Since the quantification range of plasma viral load assays has evolved over time, for analytical purposes, we truncated our measurements to range from <50 (coded as 49) to >100,000 (coded as 100,010) copies/mL. CD4 cell counts were measured by flow cytometry, followed by fluorescent monoclonal antibody analysis (Beckman Coulter, Inc., Mississauga, ON, Canada). The CD4 data come from different laboratories across BC, and in our database we capture >85% of all CD4 tests done in the province. In the Supplementary file, we include a sub-analysis assessing the difference between the populations included and excluded from this study.

Data were stratified by age measured at cART initiation (20 to 24, 25 to 44, 45 to 64, ≥65 years), gender (male and female), history of injection drug use (no, yes, unknown), era defined according to the year of first cART (2003 to 2005, 2006 to 2008, 2009 to 2012), baseline CD4 cell count (<50, 50 to 199, 200 to 349, ≥350 cells/mm^3^), baseline plasma viral load (<5 log_10_ copies/mL, ≥5 log_10_ copies/mL), adherence level measured during the first year on cART (<40, 40 to <80, 80 to <95, ≥95%) and by whether individuals achieved viral suppression within nine months of the initial cART date. Viral suppression was defined by two consecutive plasma viral loads of less than 50 copies/mL. In the Supplementary file, we include a sub-analysis assessing the difference between PLHIV classified as suppressed and unsuppressed based on this conservative definition. Adherence was defined as the number of days’ worth of antiretrovirals dispensed divided by the number of days of follow-up (expressed as a percentage) [14,23,24]. cART eras were stratified based on different treatment expansion periods in BC since 2003. From 2003 to 2008, cART expansion was directly related to treatment eligibility criteria and outreach programs to maximize engagement of PLHIV on treatment. Between 2009 and 2012, the BC government funded a targeted HIV testing, care and support outreach initiative (STOP HIV/AIDS) aimed at the two worst-affected regions in the province [11]. This initiative expanded to the entire province in 2012. BC is in an ideal position to implement this strategy given its publicly funded healthcare system that fully subsidizes access to medical services, laboratory monitoring and to cART with no co-payments or deductibles.

The size of the general population was obtained from BC Stats, which is the central statistical governmental agency for the province [25]. All-cause mortality data were obtained from the BC Vital Statistics Agency for PLHIV and the total population [26]. Deaths occurring among those living with HIV during the follow-up period were identified on a continuous basis from physician reports and through monthly record linkages carried out with the BC Vital Statistics Agency. For the general population, the BC Vital Statistics Agency only had published data up to 2011. Therefore, all analyses comparing outcomes between PLHIV and the general population only included data from January 1, 2003, to December 31, 2011. These data were also stratified by age group (20 to 24, 25 to 44, 45 to 64, ≥65 years), gender and cART era (2003 to 2005, 2006 to 2008, 2009 to 2011).

### Study outcomes and statistical methods

Crude, era-, gender- and age-specific all-cause mortality rates (per 1000 person-years of follow-up) were obtained for the general population and for those PLHIV who had started treatment. These mortality rates were calculated dividing the number of deaths (all causes) by the number of person-years of follow-up; corresponding 95% confidence intervals (CIs) for these rates were based on the Fisher's exact test [27]. Person-years for PLHIV on treatment were calculated from the date of first cART to the date of death, the last contact date (i.e. the date for a laboratory test, a prescription refill or a physician visit) or December 31, 2013, whichever came first. Person-years for the general population were calculated based on the size of the BC population for a given year. In this case, for each era, we assumed that the size of the population corresponded to the average population in each year in the era. Lastly, we calculated the person-years by multiplying the number of years by the population in each of the eras.

Standardized mortality ratios (SMRs) were calculated to compare the era- and gender-specific mortality rates between the BC population and the PLHIV in our cohort. SMRs were calculated as outlined in Rothman and Greenland [28] and 95% CIs were based on the Fisher's exact test [27]. We also conducted sensitivity analyses by stratifying the SMRs by different variables of interest to assess whether there was a sub-population in this cohort that had mortality rates approaching that of the general BC population. Abridged life tables were constructed from age-specific mortality rates to estimate the life expectancy at age 20 years for PLHIV in our cohort. The construction of these life tables and their respective 95% CIs were obtained as outlined in Chiang [29]. Categorical variables were compared using the Fisher's exact test (for 2×2 tables) or the Cochran-Mantel-Haenszel test (for other table sizes), and continuous variables were compared using the Wilcoxon rank-sum test [27]. All analyses were performed using SAS software version 9.3 (SAS, Cary, NC, USA).

The BC-CfE received approval for this study from the University of British Columbia ethics review committee at the St Paul's Hospital, Providence Health Care site (P05–123). The study complies with the BC Freedom of Information and Protection of Privacy Act. The study was conducted primarily using anonymized administrative databases, and therefore informed consent was not required.

## Results

**Figure 1:**
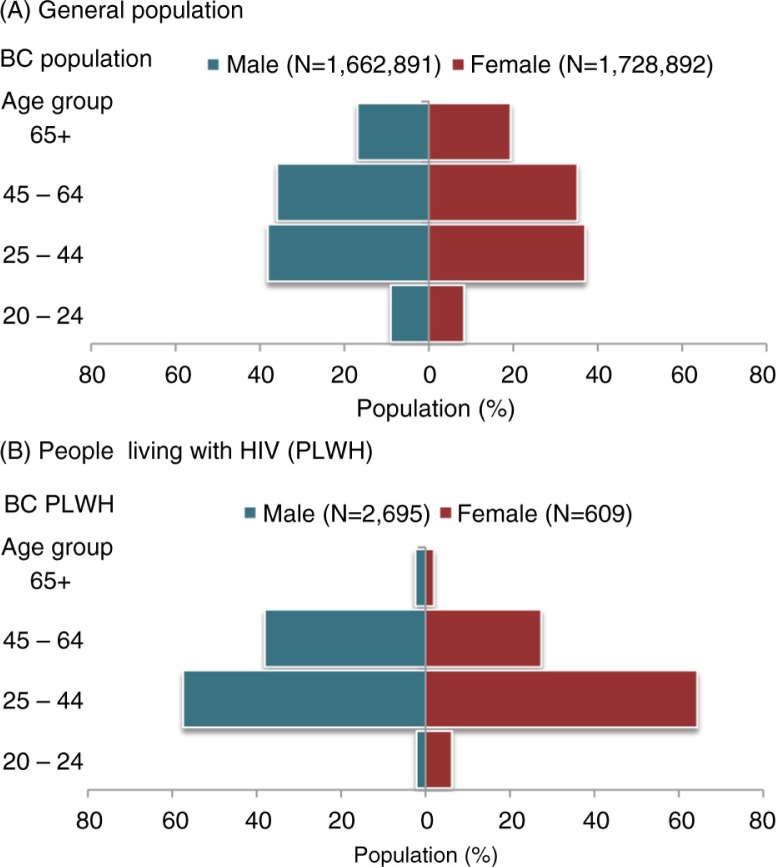
Age and gender distribution of the British Columbia general population and people living with HIV who had initiated combination antiretroviral therapy between 2003 and 2011.

Our study was based on 3653 PLHIV who initiated cART for the first time between 2003 and 2012. Figure 1 compares the age and gender structure of the general population and of the HIV-positive population who had started cART. Note that in this figure follow-up was restricted to 2003 to 2011, given the data availability for the BC general population. Based on this figure, the HIV-positive population for both genders in the age group ≥65 years and for males in the age category 20 to 24 years were highly underrepresented in comparison to the general population. In contrast, it is noticeable that the PLHIV population had the highest proportion of people, especially females, in the 25 to 44 years age category in comparison to the general population.

Table 1 compares the mortality rate and SMRs between the general population and PLHIV who had started therapy. The overall SMR was 3.22 (95% CI 2.94 to 3.52), showing that after adjusting for the effect of differences in age and gender structure between these populations, there is still considerable excess in mortality among PLHIV in comparison to the general BC population. The SMR for males was 2.96 (95% CI 2.67 to 3.27) and for females it was 4.02 (95% CI 3.29 to 4.86). This table also shows that the gap in mortality is decreasing over time for both genders, however more rapidly among females.

**Table 1:** Comparison of mortality rates and standardized mortality ratios between the general population and people living with HIV in British Columbia, 2003 to 2011

			Population		Deaths		Mortality rate per 1000 person-years (95% CI)		
					
Gender	Era	Age (years)	General population (average)	HIV+ treated population	General population	HIV+ treated population	General population	HIV+ treated population	SMR 95% CI
Male	2003 to 2011	≥20	1,662,891	2695	139,552	382	9.32 (9.28 to 9.37)	27.58 (24.88 to 30.49)	2.96 (2.67 to 3.27)
Female	2003 to 2011	≥20	1,728,892	609	133,754	106	8.60 (8.55 to 8.64)	34.57 (28.3 to 41.82)	4.02 (3.29 to 4.86)
Male	2003 to 2005	≥20	1,584,187	433	44,703	169	9.41 (9.32 to 9.49)	32.24 (27.56 to 37.48)	3.43 (2.93 to 3.98)
	2006 to 2008	≥20	1,658,462	841	47,210	148	9.49 (9.40 to 9.58)	28.42 (24.03 to 33.39)	3.00 (2.53 to 3.52)
	2009 to 2011	≥20	1,746,025	1421	47,639	65	9.09 (9.01 to 9.18)	19.11 (14.75 to 24.35)	2.10 (1.62 to 2.68)
Female	2003 to 2005	≥20	1,652,187	99	43,026	47	8.68 (8.60 to 8.76)	40.67 (29.88 to 54.08)	4.68 (3.44 to 6.23)
	2006 to 2008	≥20	1,726,497	181	45,186	48	8.72 (8.64 to 8.81)	43.43 (32.02 to 57.58)	4.98 (3.67 to 6.60)
	2009 to 2011	≥20	1,807,994	329	45,542	11	8.40 (8.32 to 8.47)	13.66 (6.82 to 24.45)	1.63 (0.81 to 2.91)
Overall			3,391,784	3304	273,306	488	8.95 (8.92 to 8.99)	28.85 (26.34 to 31.52)	3.22 (2.94 to 3.52)

*Note*: All people living with HIV included in the study had initiated combination antiretroviral therapy; data stratified by gender and treatment initiation era. Standardized mortality ratio (SMR): HIV+/general population. CI, confidence interval.

Table 2 summarizes the clinical and demographic characteristics of PLHIV in BC. The majority of individuals were male (82%), were between 25 and 44 years of age (59%), had no history of injection drug use (45%), started cART in 2009 to 012 (46%), had a baseline CD4 cell count between 50 and 349 cells/mm^3^ (64%) and had a baseline plasma viral load less than 5.00 log_10_ copies/mL (58%). The median follow-up of these individuals was 4.5 years (25th to 75th percentile (Q1 to Q3): 2.4 to 6.8 years). Additionally, 67% of individuals had adherence levels ≥95% during the first year on cART and 70% achieved viral suppression within nine months from the start of therapy.

**Table 2:** All-cause mortality rates and life expectancies for people living with HIV in British Columbia, 2003 to 2012

	Population size	Observed deaths	Follow-up (person-years)	Mortality rate (95% CI) per 1000 person-years	Life expectancy at age 20 years (standard error)
Overall	3653	498	17,430	28.57 (26.12 to 31.19)	34.53 (1.09)
Overall after one year of follow-up	3406 (93%)	316	17,329	18.24 (16.28 to 20.36)	48.70 (1.30)
Age (years)					
20 to 24	109 (3%)	6	473	12.68 (4.66 to 27.61)	NA
25 to 44	2138 (59%)	246	10,264	23.97 (21.07 to 27.16)	
45 to 64	1324 (36%)	221	6338	34.87 (30.42 to 39.78)	
≥65	82 (2%)	25	355	70.41 (45.57 to 103.96)	
Gender					
Male	2979 (82%)	389	14,268	27.26 (24.62 to 30.11)	37.48 (1.21)
Female	674 (18%)	109	3163	34.46 (28.30 to 41.57)	27.23 (1.93)
History of injection drug use					
No	1633 (45%)	134	8105	16.53 (13.85 to 19.58)	50.74 (1.73)
Yes	1309 (36%)	278	6463	43.01 (38.11 to 48.38)	24.60 (1.46)
Unknown	711 (19%)	86	2862	30.05 (24.04 to 37.11)	32.73 (2.52)
cART era					
2003 to 2005	833 (23%)	216	6398	33.76 (29.41 to 38.57)	28.82 (1.82)
2006 to 2008	1151 (32%)	196	6312	31.05 (26.86 to 35.72)	20.76 (1.91)
2009 to 2012	1669 (46%)	86	4720	18.22 (14.57 to 22.50)	49.81 (1.69)
CD4 cell count (cells/mm^3^)					
<50	421 (12%)	100	2130	46.95 (38.20 to 57.10)	22.13 (2.71)
50 to 199	1154 (32%)	233	6366	36.60 (32.05 to 41.61)	28.81 (1.52)
200 to 349	1172 (32%)	119	5851	20.34 (16.85 to 24.34)	48.47 (2.84)
≥350	906 (25%)	46	3083	14.92 (10.92 to 19.90)	53.14 (2.70)
Viral load (log_10_ copies/mL)					
<5.00	2108 (58%)	241	9458	25.48 (22.37 to 28.91)	37.87 (1.43)
≥5.00	1545 (42%)	257	7972	32.24 (28.42 to 36.43)	31.09 (1.67)
Adherence to therapy during the first year on cART (%)					
0 to <40%	220 (6%)	75	938	80.00 (62.89 to 100.23)	16.42 (1.78)
40 to <80%	468 (13%)	113	1984	56.96 (46.94 to 68.48)	17.62 (1.95)
80 to <95%	501 (14%)	66	2196	30.05 (23.24 to 38.24)	33.80 (2.46)
≥95%	2464 (67%)	244	12,312	19.82 (17.41 to 22.47)	47.26 (1.36)
Suppression at nine months					
Yes	2560 (70%)	203	12,901	15.73 (13.64 to 18.06)	54.52 (1.31)
No	658 (18%)	56	3728	15.02 (11.35 to 19.51)	30.08 (1.97)

*Note*: All people living with HIV included in the study had initiated combination antiretroviral therapy (cART); data stratified by several demographic and clinical characteristics. CI, confidence interval; NA, not applicable.

At the end of follow-up, we observed 498 deaths for a crude mortality rate of 28.57 per 1000 person-years (95% CI 26.12 to 31.19) and corresponding life expectancy (at the age of 20 years) of 34.53 years (standard error (SE) 1.09) (Table 2). If we restricted these measures to only those individuals alive after the first year (67%), the mortality rate dropped to 18.24 person-years (36% reduction; 95% CI 16.28 to 20.36) and their life expectancy increased to 48.70 years (41% increase; SE 1.30).

Table 2 also shows the mortality rates and life expectancies (at age 20 years) stratified by several individual and clinical characteristics. Mortality rates improved substantially according to cART era, ranging from 33.76 (95% CI 29.41 to 38.57) in 2003 to 2005 to 18.22 (95% CI 14.57 to 22.50) in 2009 to 2012; these changes corresponded to increases in life expectancy over these eras from 28.82 years (SE 1.82) to 49.81 years (SE 1.69). There were also important differences in mortality rate and life expectancy by history of injection drug use, CD4 cell count and plasma viral load at cART initiation, by whether these individuals achieved viral load suppression nine months after cART initiation and by whether they survived the first year on cART.

Table 3 presents factors that potentially explain the differences in mortality outcomes over time. The CD4 cell count distribution of individuals initiating cART has changed dramatically, from most individuals initiating in 2003 to 2005 at a CD4 cell count <200 cells/mm^3^ to the most recent period initiating at a CD4 cell count ≥350 cells/mm^3^. In 2009 to 2012, most PLHIV initiated therapy with a lower viral load level (*p*<0.0001) and with adherence levels in the category 80 to <95% (*p*<0.0001). It is interesting to show that in 2003 to 2008 most individuals starting cART were more likely to have a history of injection drug use, and in 2009 to 2012 those initiating cART were more likely to not have such history (*p*=0.0003).

**Table 3:** Demographic and clinical characteristics of people living with HIV who initiated combination antiretroviral therapy in British Columbia between 2003 and 2012

	cART era			
	
Variable	2003 to 2005	2006 to 2008	2009 to 2012	*p*
Gender				
Male	678 (23%)	944 (32%)	1357 (46%)	0.8826
Female	155 (23%)	207 (31%)	312 (46%)	
Age (years)				
20 to 24	18 (17%)	29 (27%)	62 (57%)	0.1115
25 to 44	500 (23%)	649 (30%)	989 (46%)	
45 to 64	295 (22%)	443 (33%)	586 (44%)	
≥65	20 (24%)	30 (37%)	32 (39%)	
History of injection drug use				
No	360 (22%)	528 (32%)	745 (46%)	<0.0001
Yes	344 (26%)	452 (35%)	513 (39%)	
Unknown	129 (18%)	171 (24%)	411 (58%)	
History of injection drug use (excluding unknown)				
No	360 (22%)	528 (32%)	745 (46%)	0.0003
Yes	344 (26%)	452 (35%)	513 (39%)	
CD4 cell count (cells/mm^3^)				
<50	158 (38%)	132 (31%)	131 (31%)	<0.0001
50 to 199	393 (34%)	447 (39%)	314 (27%)	
200 to 349	225 (19%)	427 (36%)	520 (44%)	
≥350	57 (6%)	145 (16%)	704 (78%)	
Viral load (log_10_ copies/mL)				
<5.00	467 (30%)	538 (35%)	540 (35%)	<0.0001
≥5.00	366 (17%)	613 (29%)	1129 (54%)	
Adherence to therapy during the first year on cART (%)				
≥95%	554 (22%)	779 (32%)	1131 (46%)	<0.0001
80 to <95%	83 (17%)	167 (33%)	251 (50%)	
40 to <80%	123 (26%)	137 (29%)	208 (44%)	
0 to <40%	73 (33%)	68 (31%)	79 (36%)	
Follow-up (person-years)	8.81 (7.08 to 9.83)	5.94 (5.16 to 6.81)	2.81 (1.84 to 3.85)	<0.0001

*Note*: Data stratified by combination antiretroviral therapy (cART) era.

The mortality rate and life expectancy also varied substantially by gender. Females had a mortality rate of 34.46 person-years (95% CI 28.30 to 41.57) versus 27.26 (95% CI 24.62 to 30.11) for males, which corresponded to a life expectancy of 27.23 years (SE 1.93) versus 37.48 years (SE 1.21), respectively (Table 2). Factors that may explain the difference in mortality outcomes by gender are presented in Table 4. Females were more likely to be in the age group 20 to 24 years at baseline, to have a history of injection drug use and to have had adherence <40% during the first year on therapy and a viral load <5 log_10_ copies/mL at baseline (all *p*<0.05). Males, on the other hand, were more likely to be in the age group 45 to 64 years at baseline, to have no history of injection drug use, to have had adherence ≥95% during the first year on therapy and a viral load ≥5 log_10_ copies/mL at baseline (all *p*<0.05).

**Table 4:** Demographic and clinical characteristics of people living with HIV who initiated combination antiretroviral therapy in British Columbia between 2003 and 2012

	Gender		
	
Variable	Male	Female	*p*
Age (years)			
20 to 24	69 (63%)	40 (37%)	<0.0001
25 to 44	1706 (80%)	432 (20%)	
45 to 64	1135 (86%)	189 (14%)	
≥65	69 (84%)	13 (16%)	
cART era			
2003 to 2005	678 (81%)	155 (19%)	0.8826
2006 to 2008	944 (82%)	207 (18%)	
2009 to 2012	1357 (81%)	312 (19%)	
History of injection drug use			
No	1418 (87%)	215 (13%)	<0.0001
Yes	924 (71%)	385 (29%)	
Unknown	637 (90%)	74 (10%)	
History of injection drug use (excluding unknown)			
No	1418 (87%)	215 (13%)	<0.0001
Yes	924 (71%)	385 (29%)	
CD4 cell count (cells/mm^3^)			
<50	355 (84%)	66 (16%)	0.1733
50 to 199	908 (79%)	246 (21%)	
200 to 349	956 (82%)	216 (18%)	
≥350	760 (84%)	146 (16%)	
Viral load (log_10_ copies/mL)			
<5.00	1696 (80%)	412 (20%)	0.0465
≥5.00	1283 (83%)	262 (17%)	
Adherence to therapy during the first year on cART (%)			
≥95%	2124 (86%)	340 (14%)	<0.0001
80 to <95%	385 (77%)	116 (23%)	
40 to <80%	334 (71%)	134 (29%)	
0 to <40%	136 (62%)	84 (38%)	
Follow-up (person-years)	4.53 (2.44 to 6.87)	4.28 (2.43 to 6.57)	0.3990

*Note*: Data stratified by gender; cART, combination antiretroviral therapy.

Based on Table 2, life expectancies were dramatically higher for individuals who had no history of injection drug use, survived at least one year after cART initiation, those who initiated cART with a CD4 cell count ≥350 cells/mm^3^, those who achieved adherence during the first year on therapy ≥95% and among individuals who achieved viral suppression at nine months. Based on these characteristics, we present Table 5, in which we calculated the SMR between the general population and PLHIV who have started cART, stratifying by these previous factors. Based on this table, there were significant reductions in the SMR for all stratifications. However, when we only included those without a history of injection drug use, some SMRs were not statistically significant, meaning that there is no significant excess of mortality rate in the HIV-positive population in this study compared to that of the general population.

**Table 5:** Comparison of standardized mortality ratios between the British Columbia general population and people living with HIV who initiated combination antiretroviral therapy in British Columbia, 2003 to 2011

Gender	Era	Age (years)	History of injection drug use	Restriction	SMR (95% CI)
Both	2003 to 2011	≥20	Any	After one year of follow-up (*N*=3080; mortality rate=18.60)	2.08 (1.85 to 2.32)
				CD4 cell count ≥350 cells/mm^3^ (*N*=703; mortality rate=15.82)	1.77 (1.28 to 2.37)
				Adherence to therapy during the first year on cART ≥95% (*N*=2230; mortality rate=19.90)	2.22 (1.95 to 2.52)
				Achieved suppression at nine months (*N*=2305; mortality rate=15.82)	1.77 (1.53 to 2.03)
Male	2003 to 2011	≥20	Any	After one year of follow-up (*N*=2504; mortality rate=16.98)	1.82 (1.60 to 2.07)
				CD4 cell count ≥350 cells/mm^3^ (*N*=590; mortality rate=13.70)	1.47 (1.01 to 2.07)
				Adherence to therapy during the first year on cART ≥95% (*N*=1924; mortality rate=19.75)	2.12 (1.84 to 2.43)
				Achieved suppression at nine months (*N*=1940; mortality rate=15.52)	1.66 (1.42 to 1.94)
Female	2003 to 2011	≥20	Any	After one year of follow-up (*N*=576; mortality rate=25.91)	3.01 (2.39 to 3.76)
				CD4 cell count ≥350 cells/mm^3^ (*N*=113; mortality rate=26.93)	3.13 (1.62 to 5.47)
				Adherence to therapy during the first year on cART ≥95% (*N*=306; mortality rate=20.89)	2.43 (1.68 to 3.40)
				Achieved suppression at nine months (*N*=365; mortality rate=17.43)	2.03 (1.40 to 2.83)
Both	2003 to 2011	≥20	No	Overall (*N*=1452; mortality rate=16.60)	1.85 (1.55 to 2.20)
				After one year of follow-up (*N*=1382; mortality rate=10.25)	1.14 (0.91 to 1.43)
				CD4 cell count ≥350 cells/mm^3^ (*N*=322; mortality rate=11.16)	1.25 (0.70 to 2.06)
				Adherence to therapy during the first year on cART ≥95% (*N*=1108; mortality rate=12.22)	1.36 (1.08 to 1.71)
				Achieved suppression at nine months (*N*=1140; mortality rate=10.43)	1.16 (0.90 to 1.48)
Male	2003 to 2011	≥20	No	Overall (*N*=1263; mortality rate=16.80)	1.80 (1.49 to 2.16)
				After one year of follow-up (*N*=1200; mortality rate=10.40)	1.12 (0.87 to 1.41)
				CD4 cell count ≥350 cells/mm^3^ (*N*=279; mortality rate=11.99)	1.29 (0.70 to 2.16)
				Adherence to therapy during the first year on cART ≥95% (*N*=992; mortality rate=12.73)	1.37 (1.07 to 1.72)
				Achieved suppression at nine months (*N*=996; mortality rate=10.82)	1.16 (0.89 to 1.49)

*Note*: Data stratified by several factors influencing the life expectancy of those living with HIV. Standardized mortality ratio (SMR): HIV+/general population; *N*, HIV+ treated population size; mortality rate per 1000 person-years; cART, combination antiretroviral therapy; CI, confidence interval.

## Discussion

Our results demonstrated that the life expectancy of PLHIV who initiated cART in BC has dramatically increased over the last decade. This finding is consistent with improvements in the efficacy and effectiveness of these regimens and the province's direct efforts to increase the early engagement of individuals on treatment when these individuals are less likely to be seriously immune-compromised and more likely to fully benefit from therapy. We also showed that, overall, the gap in mortality between BC residents and the HIV-positive population is decreasing.

Our results are also important since they quantify the direct and significant effect of cART clinical outcomes on life expectancy. This is demonstrated by first showing that an individual's ability to suppress at nine months or to have adherence level ≥95% during the first year on therapy can increase life expectancy by more than 20 years. This is further highlighted by the fact that individuals who were engaged on treatment and survived their first year on therapy saw their life expectancy increase dramatically by nearly 15 years. In particular, we showed that individuals with high CD4 cell counts at cART baseline had an excess in life expectancy of 30 years in comparison to those with lower counts, and they had a significantly lower SMR. Given the recent results of the START trial and the fact that current treatment guidelines recommend early testing and treatment to all PLHIV regardless of CD4 cell count, our results have further confirmed the effectiveness of cART in decreasing premature mortality [30,31].

We were able to show that life expectancy and mortality rates varied substantially by gender and that the great efforts that have been in place to minimize the gap between males and females seem to have had a significant effect over time. These efforts are a collaboration of the BC-CfE, the BC government and different cohort studies currently in place, including the following: the Canadian HIV Women's Sexual and Reproductive Health Cohort Study, to better understand factors associated with treatment outcomes among females in BC and across Canada [32]; the Momentum Health Study of the various factors associated with the HIV epidemic among gay, bisexual and other men who have sex with men in the Greater Vancouver region [33]; and several different cohorts of people who use drugs and sex workers in the Greater Vancouver area, focused on identifying and understanding the many factors that affect the health of this vulnerable population [34,35].

In the last 10 years, a handful of studies have reported improvements in life expectancy for PLHIV in different settings [3–6,36,37], including two studies combining data from different cohorts across North America and Europe. Although these last studies are important to the field and in agreement with ours, it is important to highlight that the target populations were highly heterogeneous, with different mortality reporting systems and based on fairly different health systems for each of the countries considered. In contrast, our study was conducted within a fully subsidized medical system where antiretroviral therapy as well as medical and laboratory monitoring are free of charge to all study participants. Thus, our results were less likely to be biased by direct financial limitations to access to health services, a frequent confounder in cohort studies. A second strength of our study was that delayed reporting of deaths was not likely a factor to influence our results, since most deaths were reported within one month through active follow-up with physicians and regular linkages to the BC Vital Statistics Agency. In this study, since 18% of individuals were lost to follow up (LFU), there is the possibility of missing several death events and biasing our mortality estimates downwards. To address this concern, in the Supplementary file, we include a sub-analysis assessing the difference between PLHIV classified as LFU and alive at the end of follow-up. To address this problem, we would need to link our data to a national death registry to account at least for individuals who moved out of BC to other provinces. However, at this moment the Drug Treatment Program has neither access nor ethics approval to link its data to such a registry. It is worth mentioning that efforts within the STOP HIV/AIDS initiative are in place to minimize the risk of individuals being LFU and to re-engage those already LFU. Moreover, our mortality data was obtained through an ascertainment method involving active follow-up with physicians and monthly linkages with BC Vital Statistics, a province-wide institution that tracks all deaths occurring in BC. This ascertainment approach for deaths is likely to have improved the accuracy in estimating the number of deaths in the province. Finally, the centralized and population-based nature of the BC-CfE HIV/AIDS Drug Treatment Program, which monitors and evaluates individuals living with HIV health outcomes and response to cART, provides a unique opportunity for characterization of the impact of the recent scaling-up of cART on mortality patterns in BC.

Our study has several potential limitations/features that should be considered in the interpretation of our findings. Note that there was a small number of individuals in our study aged 65 year or older at the time of cART initiation, and it is important to interpret these results with caution since the mortality rates in this age group may be underestimated. Additionally, in this study, we did not fully control for differences in risk group (for HIV acquisition) since changes in data collection have led to a high proportion of individuals in our database missing their risk information. In BC, the HIV epidemic has always been concentrated among men who have sex with men and people who have injected drugs, and therefore our estimated mortality rates and associated life expectancies have included both the high and low risk populations in BC. In this study, we did not control for socio-economic variables (e.g. ethnicity, income, education) that may influence our mortality outcomes, as the data collection for these variables is deficient. Lastly, since this study was observational, we cannot assume that the associations we found were causal.

## Conclusions

Our results demonstrate that the life expectancy of PLHIV who have initiated cART has dramatically increased over the last decade with a consequent decreasing gap in SMRs between BC residents and the HIV-positive population. We also observed much lower mortality rates in both males and females and an interesting downward shift in the gender gap of these rates among PLHIV. However, there is still room for improvement and multilateral efforts should continue to promote sustained engagement of HIV-positive individuals early in treatment and minimize LFU so that the impact of cART can be fully realized.

## Supplementary Material

The impact of scaling-up combination antiretroviral therapy on patterns of mortality among HIV-positive persons in British Columbia, Canada
