# Supplementary material for: The impact of scaling-up combination antiretroviral therapy on patterns of mortality among HIV-positive persons in British Columbia, Canada
Source: J Int AIDS Soc. 2015 Oct 7;18(1):20261. doi: 10.7448/IAS.18.1.20261 (PMC4598331; doi:10.7448/IAS.18.1.20261)
Supplement: The impact of scaling-up combination antiretroviral therapy on patterns of mortality among HIV-positive persons in British Columbia, Canada [file JIAS-18-20261-s001.pdf]

Comparison of individuals excluded from the analyses to those included

A total of 743 individuals have been excluded since they did not have a viral load and/or CD4 cell count at baseline, they started combination antiretroviral therapy (cART) in a non-recommended regimen according to treatment guidelines, the cART regimen included drugs outside those eligible for this analysis, or since their treatment/monitoring history was inadequately reported in our database.

We compared the variables gender, age, era of cART initiation, history of injection drug use and follow-up, between the individuals in this study and those who were excluded. We observed that those excluded were more likely to have started cART between 2009 and 2012 (71% versus 46%; p-value <0.0001), to have unknown history of injection drug use (53% vs 45%; p-value <0.0001), to be younger (41 (25th-75th percentile [Q1-Q3] 33-49) vs 42 (Q1-Q3 35-49) years; p-value 0.0191) and to have shorter follow-up than those included in this study (0.9 (Q1-Q3 0.4-3.9) versus 4.5 (Q1-Q3 2.4-6.8) years; p-value <0.0001). These two groups did not differ by gender (p-value 0.236).

Explanation of individuals who were not classified as suppressed in this study

In this study, viral suppression was defined by two consecutive plasma viral loads of less than 50 copies/mL within nine months since cART initiation. Based on this definition, 2560 (70%) PLWH were classified as suppressed, while 658 (18%) were classified as unsuppressed. Note that 435 (12%) individuals were unclassified (i.e. received missing for this variable) since they had follow-up time during the study shorter than 9 months. Therefore, it would not be correct to assign these individuals to the unsuppressed group. For the 658 (18%) individuals classified as unsuppressed, they had at least 9 months of follow-up and:

- 38 (6%) achieved suppression after 9 months
- 81 (12%) had only one available viral load in this period
- 539 (82%) had at two consecutive viral load measurements in this period and at least one of them was above 50 copies/mL.

Comparison of individuals who were alive at the end of study and those who were lost to follow-up

In this study, 18% of individuals were classified as lost to follow-up. We compared the variables gender, age, era of cART initiation, history of injection drug use and follow-up between those who were lost to follow-up and those alive at the end of follow-up. We observed that those lost to follow-up were more likely to have no history of injection drug use (49% vs 47%; p-value <0.0001), to be younger (40 (Q1-Q3 33-47) vs 42 (Q1-Q3 35-49) years; p-value <0.0001), and to have shorter follow-up than those alive (3.26 (Q1-Q3 1.59-5.88) vs 5.0 (Q1-Q3 3.1-7.3) years p-value <0.0001). These two groups did not differ by gender (p-value 0.1124) and era of cART initiation (p-value 0.285).
